# Increase in Hospital-Acquired Carbapenem-Resistant *Acinetobacter baumannii* Infection and Colonization in an Acute Care Hospital During a Surge in COVID-19 Admissions — New Jersey, February–July 2020

**DOI:** 10.15585/mmwr.mm6948e1

**Published:** 2020-12-04

**Authors:** Stephen Perez, Gabriel K. Innes, Maroya Spalding Walters, Jason Mehr, Jessica Arias, Rebecca Greeley, Debra Chew

**Affiliations:** ^1^Epidemic Intelligence Service, CDC; ^2^Communicable Disease Service, New Jersey Department of Health; ^3^Division of Healthcare Quality Promotion, National Center for Emerging and Zoonotic Infectious Diseases, CDC; ^4^New Jersey Medical School, Rutgers University, Newark, New Jersey.

*On December 1, 2020, this report was posted online as an *MMWR* Early Release.*

Carbapenem-resistant *Acinetobacter baumannii* (CRAB), an opportunistic pathogen primarily associated with hospital-acquired infections, is an urgent public health threat (*1*). In health care facilities, CRAB readily contaminates the patient care environment and health care providers’ hands, survives for extended periods on dry surfaces, and can be spread by asymptomatically colonized persons; these factors make CRAB outbreaks in acute care hospitals difficult to control (*2*,*3*). On May 28, 2020, a New Jersey hospital (hospital A) reported a cluster of CRAB infections during a surge in patients hospitalized with coronavirus disease 2019 (COVID-19). Hospital A and the New Jersey Department of Health (NJDOH) conducted an investigation, and identified 34 patients with hospital-acquired multidrug-resistant CRAB infection or colonization during February–July 2020, including 21 (62%) who were admitted to two intensive care units (ICUs) dedicated to caring for COVID-19 patients. In late March, increasing COVID-19–related hospitalizations led to shortages in personnel, personal protective equipment (PPE), and medical equipment, resulting in changes to conventional infection prevention and control (IPC) practices. In late May, hospital A resumed normal operations, including standard IPC measures, as COVID-19 hospitalizations decreased, lessening the impact of personnel and supply chain shortages on hospital functions. CRAB cases subsequently returned to a pre–COVID-19 baseline of none to two cases monthly. The occurrence of this cluster underscores the potential for multidrug-resistant organisms (MDROs) to spread during events when standard hospital practices might be disrupted; conventional IPC strategies should be reinstated as soon as capacity and resources allow.

Hospital A is an urban, acute-care hospital in New Jersey with approximately 500 beds. In May 2020, hospital A notified NJDOH of an increase in CRAB (*A. baumannii* with meropenem minimum inhibitory concentration testing of ≥8 *μ*g/mL) isolates from weekly ICU point prevalence surveys (colonization screening) and from clinical infections. Hospital A retrospectively reviewed microbiology records for CRAB isolated from inpatient specimens since November 2019 and instituted prospective surveillance of laboratory results to identify all CRAB isolates. Inpatients with hospital-acquired CRAB infection were defined as those for whom CRAB was isolated from clinical or colonization screening specimens collected on or after hospital day 3 and who had no earlier CRAB isolated from specimens during the same hospitalization; incident CRAB was a patient‘s first CRAB infection or colonization. Patients’ demographic characteristics, diagnoses, treatments, disposition, and COVID-19 status were collected from medical records. Diagnoses of CRAB infection or colonization were determined by infectious disease specialists. NJDOH began an investigation to assess IPC practices at hospital A and gather additional data. This activity was reviewed by CDC and was conducted consistent with applicable federal law and CDC policy.*

During February–July 2020, 34 patients with hospital-acquired CRAB infection or colonization were identified, including 28 (82%) whose incident CRAB infection or colonization occurred during the facility’s surge in COVID-19 cases (March–June 2020) (Figure), and 17 (50%) who had confirmed infection with SARS-CoV-2, the virus that causes COVID-19 (Table). Twenty (59%) incident cases were identified from clinical specimens and 14 (41%) through colonization screening. Median age of patients with CRAB infection was 55 years (interquartile range [IQR] = 48–64 years), and 28 patients (82%) were admitted from home. No patients had prior documented CRAB infection or colonization. The median interval from admission to incident CRAB infection was 19 days (IQR = 11–28 days). Twenty-five (74%) patients were intubated and mechanically ventilated at the time of specimen collection; those with COVID-19 were placed in a prone position. CRAB infection was diagnosed in 20 (59%) of the 34 patients, including 14 (41%) with clinically diagnosed CRAB ventilator-associated pneumonia, four of whom had bacteremia. At the time of this report, 23 (68%) patients with CRAB infection had been discharged, 10 (29%) had died, and one remained hospitalized.

**FIGURE:**
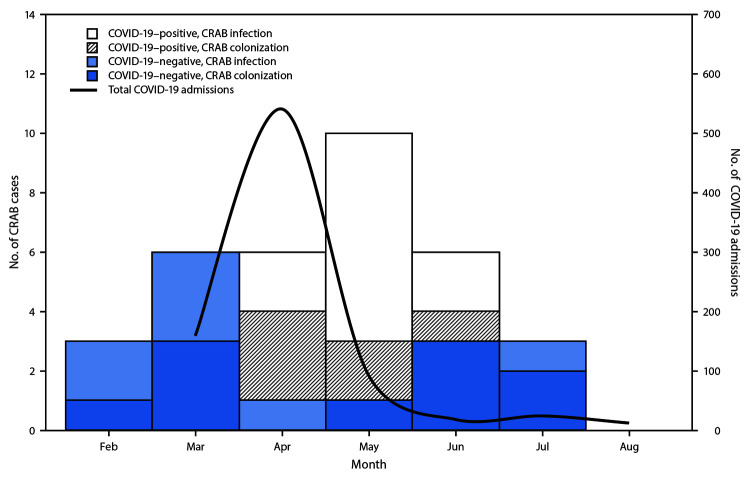
Number of admitted patients with COVID-19 (N = 846) and hospital-acquired carbapenem-resistant *Acinetobacter baumannii* (CRAB)* (N = 34), by month — hospital A, New Jersey, February–July 2020 **Abbreviation:** COVID-19 = coronavirus disease 2019. * CRAB infection or colonization.

**TABLE:** Demographic and clinical characteristics of patients with carbapenem-resistant *Acinetobacter baumannii* (CRAB) (N = 34) — hospital A, New Jersey, February–July 2020

Characteristics of patients with CRAB	No. (%) of patients
**Age, median (IQR), yrs**	55 (48–64)
**Sex**	
Male	24 (71)
Female	10 (29)
**Location before admission**	
Home	28 (82)
Skilled nursing facility	5 (15)
Long-term acute care hospital	1 (3)
**Collection location of incident CRAB**	
Intensive care unit	25 (73)
Medical-surgical unit	5 (15)
Progressive care or step-down unit	4 (12)
**Specimen source of incident CRAB**	
Respiratory (sputum, tracheal aspirate, or bronchial)	17 (50)
Axilla, groin, or rectal	6 (18)
Blood	5 (15)
Wound, bone, or other tissue	4 (12)
Urine	2 (5)
**SARS-CoV-2 status**	
Positive	17 (50)
Negative	17 (50)
**CRAB infection/colonization**	
Ventilator-associated pneumonia	10 (29)
Ventilator-associated pneumonia with bacteremia	4 (12)
Bacteremia	3 (9)
Bone or soft tissue infection	3 (9)
Colonization	14 (41)
**Intubation/Mechanical ventilation at time of incident CRAB**	
Yes	25 (74)
No	7 (21)
**Tracheostomy**	
Yes	8 (24)
No	26 (76)
**Received respiratory therapy services**	
Yes	28 (82)
No	6 (18)
**Disposition**	
Discharged/Transferred	23 (68)
Deceased	10 (29)
Remains hospitalized	1 (3)

**Abbreviation:** IQR = interquartile range

The multidrug-resistant CRAB definition (*A. baumannii* with documented resistance to three or more classes of antibiotics) was applied to hospital clinical laboratory antimicrobial susceptibility data for incident cases (*4*); all 34 met multidrug-resistant CRAB criteria. Thirty isolates were further evaluated for carbapenemase genes through real-time polymerase chain reaction testing.^†^ Twenty-six isolates harbored the gene encoding the OXA-23 carbapenemase. Among these isolates, two from specimens collected in February and March harbored an additional carbapenemase gene, encoding New Delhi metallo-β-lactamase (a gene rarely present in CRAB isolates from patients in the United States), indicating that at least one CRAB introduction occurred before the surge of COVID-19 cases (*5*). Four specimens were nonviable or did not yield CRAB growth.

During March–August 2020, hospital A admitted approximately 850 patients with COVID-19. The number of cases peaked on April 9, with 36 new hospitalizations and 61% of the inpatient census having a diagnosis of confirmed or suspected COVID-19. Pandemic-related resource challenges necessitated intentional changes to IPC measures. Before the pandemic, ventilator circuits and suctioning catheters were changed at specified intervals of every 14 days and every 3 days, respectively, unless malfunctioning or visibly soiled. To conserve equipment during the surge, the hospital’s respiratory therapy unit instituted a policy to extend the use of ventilator circuits and suctioning catheters for individual patients, replacing them only if they were visibly soiled or malfunctioning. To conserve PPE, gown use as part of Contact Precautions^§^ was suspended for care of patients with the endemic MDROs vancomycin-resistant *Enterococcus* spp. and methicillin-resistant *Staphylococcus aureus*^¶^ but was maintained for nonendemic MDROs such as CRAB. Gowns and gloves continued to be used for all patients when indicated for Standard Precautions, including wearing a gown when skin or clothing was likely to be exposed to blood or body fluids.** Anticipating shortages, hospital A also adopted an extended-use PPE protocol for N95 respirators and face shields. To prioritize personnel resources, activities of the MDRO workgroup, a multidisciplinary team responsible for guiding IPC policy around MDRO prevention efforts at hospital A, were suspended, along with biweekly bedside central venous catheter and indwelling urinary catheter maintenance rounds. Routine audits of appropriate PPE use, hand hygiene compliance, and environmental cleaning were also temporarily discontinued.

Responding to COVID-19–related care needs also resulted in other unintentional changes in standard practices for preventing the spread of MDROs and device-associated infections. IPC leadership noted less frequent patient bathing with chlorhexidine gluconate and a 43% reduction in ICU CRAB screening tests. These changes resulted from competing clinical priorities, challenges in personnel availability, and an effort to minimize staff members’ interaction time with patients. The facility experienced critical shortages in personnel for nursing and environmental services, resulting from staff members’ illness, quarantine, and a surge in the number of patients with COVID-19. Nursing resources were supplemented through agency and government entities; however, increased patient-to-staff member ratios and the need to minimize patient contact might have led to unidentified IPC breaches.

In early May, hospital A’s IPC leadership advised physicians, unit managers, and environmental services of the CRAB cluster. Environmental services cleaned common areas and high-touch surfaces of ICUs with bleach. Proper hand hygiene and PPE use were reinforced through unit-based education, and compliance audits were restarted by mid-May. At the end of May, environmental services terminally cleaned and disinfected the COVID-19 dedicated ICUs and associated portable medical and respiratory equipment. IPC personnel and unit leadership reinforced CRAB surveillance culture protocol adherence.

## Public Health Response

In collaboration with hospital A, NJDOH investigated the cluster, including review of laboratory data, patient information, IPC policies, and audit tools. NJDOH provided technical guidance on IPC interventions and advised returning to normal operations as soon as capacity allowed. IPC processes and interventions developed in collaboration with NJDOH (adapted from CDC guidelines^††^) during a previous CRAB outbreak at hospital A helped establish metrics for baseline incident case counts and adherence to IPC-related measures.

In June, NJDOH used New Jersey’s public health notification system^§§^ to alert public health officials, health care providers, and infection preventionists to the possible resurgence of MDROs in health care facilities facing COVID-19–related resource limitations. In June 2020, hospital A reported fewer incident hospital-associated CRAB cases, coinciding with a sharp decrease in COVID-19 hospitalizations (Figure). This trend continued through July. In August, no incident hospital-associated CRAB cases were reported, signaling a return to baseline numbers for the facility.

## Discussion

The impact of the COVID-19 pandemic on the spread of antibiotic resistance in health care settings has not been fully described. In response to a rapid increase in SARS-CoV-2 infections, many health care facilities adopted mitigation strategies to contend with physical space limitations, constrained availability of personnel, shortages in PPE, and a large number of critically ill patients. Recent single-facility reports from the United States and Europe have described increased acquisition of MDROs among patients hospitalized with COVID-19 (*6*–*8*). Hospital A experienced a large multidrug-resistant CRAB outbreak, primarily involving ICU patients, which extended across multiple units during a surge in COVID-19 cases.

Outbreaks of CRAB have been well documented in acute care hospitals, particularly among critically ill patients, and are often driven by factors that include breaches in infection control and persistent environmental contamination (*3*,*9*). Containing these outbreaks often requires multiple, targeted interventions, including increased surveillance, IPC audits, and environmental cleaning (*10*). During COVID-19 preparations and the ensuing surge in cases, decreased vigilance for control of CRAB transmissions, including suspension of the MDRO workgroup, reduced surveillance cultures, reduced personnel numbers (which decreased capacity for overall auditing practices), and both intentional and unintentional changes in IPC practice likely contributed to this CRAB cluster. The lack of audits made identifying and correcting real-time IPC compliance issues difficult. Diminished colonization screening might have resulted in a higher threshold for recognizing increasing incident hospital-acquired CRAB cases. Reinstatement of conventional IPC strategies in ICUs, paired with enhanced cleaning procedures and hand hygiene reeducation, likely contributed to the rapid decline in cases.

The findings in this report are subject to at least three limitations. First, CRAB can colonize persons for long periods, possibly leading to misclassification of some cases present at admission as hospital-acquired cases; decreased ICU surveillance testing might have contributed to this misclassification. Second, objective assessment of hand hygiene, PPE use, and environmental cleaning during the surge in COVID-19 cases is difficult without routine audit data. Finally, whole genome sequencing to determine the relatedness of isolates was not performed. Carbapenem resistance mechanism testing indicated at least two introductions of CRAB, including one preceding the surge. Whether OXA-23 CRAB spread into distinct patient populations (i.e., patients with and without COVID-19) or these were different introductions remains unclear.

The COVID-19 pandemic has required hospitals to take unprecedented measures to maintain continuity of patient care and protect health care personnel from infection. This outbreak highlights that MDROs can spread rapidly in hospitals experiencing surges in COVID-19 cases and cause serious infections in this setting. To reduce spread of MDROs and the risk of infection for patients, hospitals should remain vigilant to prevent and detect clusters of unusual infections and respond promptly when they are detected. Facilities should prioritize continuity of core IPC practices (e.g., training for and auditing of hand hygiene, PPE use, and environmental cleaning) to the greatest extent possible during surges in hospitalizations and make every effort to return to normal operating procedures as soon as capacity allows.

**Summary**

**What is already known about this topic?**

Carbapenem-resistant *Acinetobacter baumannii* (CRAB) causes health care–associated infections that are challenging to contain and often linked to infection prevention and control (IPC) breaches.

**What is added by this report?**

A New Jersey hospital reported a cluster of 34 CRAB cases that peaked during a surge in COVID-19 hospitalizations. Strategies to preserve continuity of care led to deviations in IPC practices; CRAB cases decreased when normal operations resumed.

**What are the implications for public health practice?**

Hospitals managing surges of patients with COVID-19 might be vulnerable to outbreaks of multidrug-resistant organism (MDRO) infections. Maintaining IPC best practices (e.g., MDRO surveillance and hand hygiene and environmental cleaning audits) to the extent possible could mitigate spread.

## Footnotes

* 45 C.F.R. part 46, 21 C.F.R. part 56; 42 U.S.C. Sect. 241(d); 5 U.S.C. Sect. 552a; 44 U.S.C. Sect. 3501 et seq.

† Real-time polymerase chain reaction testing for carbapenemase genes was performed at the Clinical Laboratory Improvement Amendments (CLIA)–certified Northeast Regional Antimicrobial Resistance Laboratory located at the Wadsworth Center at the David Axelrod Institute in Albany, New York.

†† https://www.cdc.gov/infectioncontrol/guidelines/mdro/index.html.

§§ https://www.njlincs.net/PublicHealthAlertMessages/messageviewer.aspx?id=110407.
